# Vegetation management shapes arthropod and bird communities in an African savanna

**DOI:** 10.1002/ece3.9880

**Published:** 2023-03-08

**Authors:** Dan M. Parker, Keenan Stears, Terence Olckers, Melissa H. Schmitt

**Affiliations:** ^1^ School of Biology and Environmental Sciences University of Mpumalanga Nelspruit South Africa; ^2^ Department of Ecology, Evolution, and Marine Biology University of California Santa Barbara Santa Barbara California USA; ^3^ School of Life Sciences University of KwaZulu‐Natal Scottsville South Africa

**Keywords:** avifauna, conservation, diversity, habitat management, insect, mowing, predator–prey, tree clearing

## Abstract

Habitat heterogeneity is a key driver of the diversity and distribution of species. African savannas are experiencing changes in their vegetation structure causing shifts towards increased woody plant cover, which results in vegetation structure homogenization. Given the impact that increasing woody plant cover has on patterns of animal use, resource managers across Africa are implementing habitat management practices that are intended to reduce woody plant cover. To understand the ecological implications of various habitat management practices on arthropod and bird communities, we leveraged large‐scale tree clearing and subsequent mowing in an African savanna to understand how changes in both the herbaceous layer and woody plant cover (i.e., structural heterogeneity) may shape arthropod and bird communities at the local scale. We focused on four replicated treatments: (1) annual summer mow, (2) annual winter mow, (3) >5 years since last mow (rest), and (4) an adjacent unmanipulated savanna to act as a control. We found that the mowing treatments significantly influenced vegetation structure both with respect to tree density and herbaceous layer. Both arthropod and bird community composition varied across treatments. Grass biomass was the best predictor of arthropod richness and abundance, with arthropods selecting for areas with high biomass. Insectivorous bird richness and abundance was driven by tree density (i.e., perching locations) and not arthropod abundance. Our results suggest that vegetation management practices contribute to habitat heterogeneity at the landscape scale and increase bird species richness through species turnover. However, we caution that if a single vegetation management practice dominates the landscape, it is plausible that it could lead to the simplification of the avian community.

## Graphical abstract

Habitat heterogeneity is a key driver of the diversity and distribution of species. African savannas are experiencing changes in their vegetation structure causing shifts towards increased woody plant cover, which results in vegetation structure homogenization. Given the burgeoning need to implement vegetation management practices to combat increasing woody plant cover, our results demonstrate the importance of understanding the outcomes of a similar management approach (i.e., mowing) that is applied at different temporal scales (i.e., annual summer mow, annual winter mow, >5‐year gap since last mow). We conclude that summer and winter mowing are not interchangeable and management practices that reduce grass biomass and tree density will negatively affect arthropod and bird communities.

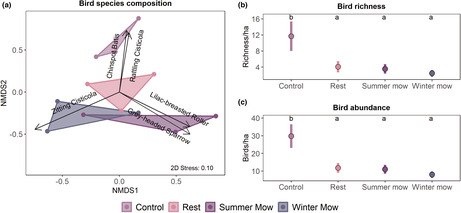

## 1 INTRODUCTION

The structural heterogeneity of vegetation is an important organizing principle in many ecosystems and has been recognized as an important driver of the diversity and distribution of species (Tews et al., 2004). Heterogeneity provides organisms with physical refugia to avoid predators (Gorini et al., 2012) as well as provide a greater variety and abundance of important resources (Atuo & O'Connell, 2017). As such, the concept of heterogeneity is central to conservation efforts (Pickett, 1997). Savanna ecosystems are heterogeneous and are characterized by the co‐dominance of trees and grasses, which are maintained by a complex set of interacting biotic and abiotic factors including geology, fire, precipitation, competition, and herbivory (Levick et al., 2009). However, due to both local (e.g., fire management) and global (e.g., increases in CO_2_ emissions) drivers, savannas are experiencing rapid and widespread increases in woody plant cover shifting them towards more woody‐dominated states (Buitenwerf et al., 2012; O'Connor et al., 2014; Stevens et al., 2016). This shift can result in vegetation homogenization (i.e., a loss in structural heterogeneity) at both regional and landscape scales (McCleery et al., 2018).

The reduction in structural heterogeneity from woody plant densification has profound impacts on animal communities, resulting in species turnover and reduced diversity in birds, bats, and other small and large mammals (McCleery et al., 2018; Schmitt et al., 2022; Sirami et al., 2009; Smit & Prins, 2015). Such changes are likely to influence the species‐driven processes that regulate ecosystem function (Pessarrodona et al., 2019) and may ultimately affect the stability and resilience of African savannas (Cromsigt & Olff, 2006; Gagic et al., 2015). Because of the effect that increasing woody plant cover (and concomitant reductions in grass cover/height) has on animal communities (Schmitt et al., 2022; Sirami et al., 2009), land managers across Africa, and globally, are implementing habitat management practices aimed to reduce the rate of increasing woody plant cover (Schmitt et al., 2022). In addition, due to the rapid rate of increase in woody encroachment (Stevens et al., 2017), land managers often resort to aggressive measures to reduce woody plant cover. Such practices include tree clearing and subsequent annual mowing of the herbaceous layer, mechanical crushing of vegetation (i.e., “mastication”), roller‐chopping, and tree thinning (Newman et al., 2018; Schmitt et al., 2022).

Vegetation structure is a key factor that drives both bird species assemblages (MacArthur & MacArthur, 1961; Sirami et al., 2009) and arthropod abundance (Dennis et al., 2007). For example, bird community composition differs between different management approaches (Brüggeshemke et al., 2022; Herremans, 1998; Kaphengst & Ward, 2008; Krook et al., 2007). Thus, through their effects on woody plant cover and the herbaceous layer, vegetation management, such as tree clearing with subsequent mowing, is likely to impact both bird and arthropod communities. In savanna ecosystems, arthropod abundance during the wet summer periods are important food sources for both resident and migratory insectivorous bird species (Little et al., 2013; Nkwabi et al., 2011). However, while several studies have assessed how vegetation management influences arthropod and bird communities independently, relatively few studies have assessed how vegetation management influences arthropod and bird communities simultaneously in savanna ecosystems (but see, Nkwabi et al., 2011). This lack of simultaneous study is surprising given the link between declining bird populations and reduced arthropod abundance (Robinson & Sutherland, 2002; Vickery et al., 2001).

To understand how changes in both the herbaceous layer and woody plant cover (i.e., structural heterogeneity) from vegetation management practices shape arthropod and bird communities, we leverage large‐scale, spatially replicated habitat management areas in an African savanna. These areas were cleared of woody vegetation >50 years ago and have been mowed annually since to prevent woody recruitment. Within each management area, large‐scale plots are mowed in either the wet (summer) or the dry (winter) season annually or have been rested from mowing for >5 years since annual vegetation management. We contrast the effect of tree clearing and the three mowing approaches against control areas in the adjacent savanna vegetation, which reflect the natural vegetation of the greater study area and has not received any mowing or tree clearing. This spatially replicated experimental design (i.e., control, rest, summer mow, and winter mow) allowed us to determine how tree‐clearing and subsequent mowing approaches influence: (1) woody and herbaceous vegetation structure, (2) arthropod community composition and abundance, (3) bird community composition and abundance, and (4) the relative importance of vegetation structure versus food availability in driving observed patterns.

We expected that management‐induced changes in vegetation would influence arthropod and bird communities. Specifically, we expected that richness and abundance metrics would follow the heterogeneity hypothesis (Huston, 1979) such that they would increase with variation in vegetation structure. Furthermore, we predicted that the composition of the arthropod and bird communities would differ between the different management treatments due to niche partitioning associated with habitat and/or prey availability. Additionally, we predicted that the various mowing approaches would likely result in species turnover spatially, with unique bird species being found in each treatment because of species traits relating to their life histories. Finally, we predicted that vegetation structure would be the driver of arthropod and bird communities, rather than food availability. Specifically, arthropods should select for treatments where vegetation structure provides refugia from predators (Prather & Kaspari, 2019), whereas birds should select for treatments where vegetation structure (i.e., perching locations) increase their hunting success (Seymour & Dean, 2009).

## 2 MATERIALS AND METHODS

### 2.1 Study site

**FIGURE 1:**
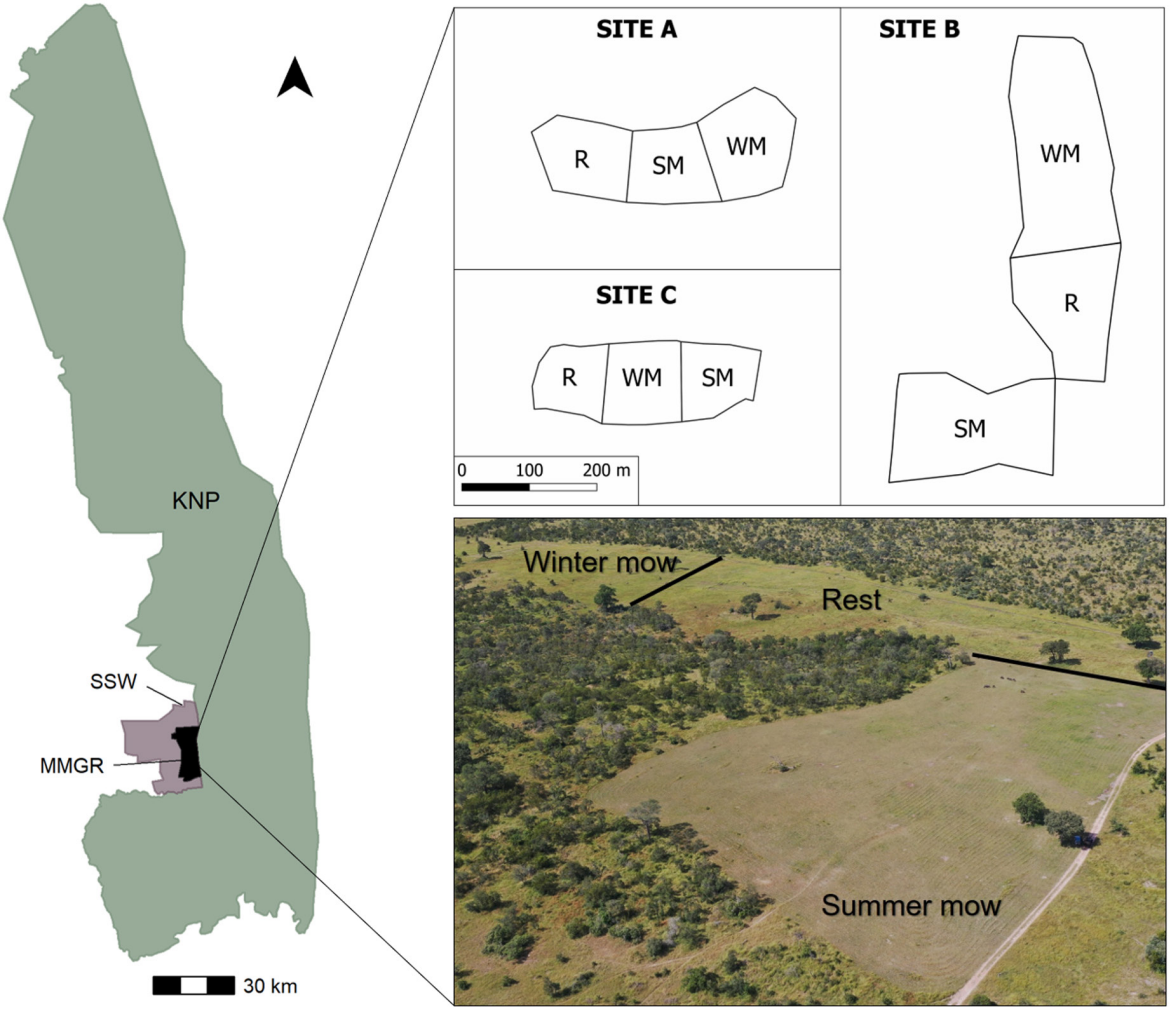
Location of our study site, MalaMala Game Reserve (MMGR), South Africa, that lies between the Sabi Sands Wildtuin (SSW) and the Greater Kruger National Park (KNP). The inset shows the randomized design of our treatments (i.e., summer mow [SM], winter mow [WM], and rest [R] within each site). The controls are located within the natural savanna vegetation adjacent to each site. The photograph depicts an aerial image of one site (Site B).

We conducted our study on MalaMala Game Reserve (13,300 ha), South Africa, during the wet season (February) of 2022. MalaMala Game Reserve falls within the Sabi Sands Wildtuin–MalaMala–Sabi Game Reserve Complex and shares an unfenced border with Kruger National Park to the east and therefore forms part of the Greater Kruger National Park (>20,000 km^2^) protected ecosystem (Figure 1). The region's mean annual rainfall is approximately 620 mm with summer rainfall occurring between October and March (Schulze, 2008). The natural vegetation at our study site is characterized by a mixed Combretum/Terminalia woodland (Gertenbach, 1983).

### 2.2 Vegetation management and experimental design

Large‐scale tree clearing was conducted at select locations at our study site in the 1960s–1970s to create grass‐dominated areas with low‐to‐no tree cover (<5 trees/ha). These tree‐cleared areas were maintained with low‐to‐no tree cover using annual mowing until 2015. In 2019, three replicate tree‐cleared management areas were selected and three different mowing treatments (i.e., summer mow, winter mow, and rest) were applied in a randomized block design, hereafter referred to as treatment plots (Figure 1). The application of the mowing treatments has been continued annually since 2019 (i.e., for 3 years at the time of our study). Summer mow treatments are mowed early in the wet season (late December–mid January), winter mow treatments are mowed at the onset of the dry season (mid–late June), and the rest treatment has remained unmowed since its last mow in 2015. At each of the three sites, adjacent savanna areas acted as unmanipulated control treatments. Given that the size of each area cleared of trees varied across the three sites (ranging from ~3.5 to 10.5 ha), the treatment blocks within a site also varied (~1–4 ha). Within a site, all treatment plots are adjacent to each other (Figure 1). All data collection, outlined below, occurred during the wet season (i.e., summer) approximately 1.5 months after the implementation of the summer mow. The winter mows were implemented in the previous dry season (i.e., winter) approximately 8 months before the start of the study.

At each site, we measured the following vegetation structural components during the wet season of February 2022: tree density (number of trees/ha), grass height (cm), grass biomass (tons/ha), and the variation in height of the herbaceous layer (as measured by the coefficient of variation of grass height). In each treatment, we established three transects that were either 80 or 100 m in length depending on the size of the treatment plot (see above). Every 1 m along these transects, we took three measurements of grass height and every 2 m along the transect, we took two measurements of grass biomass using a disc pasture meter (Trollope & Potgieter, 1986). The disk pasture meter readings were converted to estimated biomass (tons/ha) using calibrated estimates (Zambatis et al., 2006). As per Stears and Shrader (2020), we used the grass height measurements to calculate the coefficient of variation (CV = (standard deviation/mean)) of grass sward height for each treatment as a measure of the variation in grass height. Due to the low density of trees in the mow plots, it was not suitable to measure tree density using the above transect method. As such, we estimated tree density using on‐the‐ground counts where all trees within the sampling area were enumerated. Following McCleery et al. (2018) and Blaum et al. (2009), we defined a tree as any woody vegetation taller than 0.5 m in height. We repeated all the above vegetation sampling in the adjacent control savannas. To limit the influence of tree density on bird detectability (see below), we selected control areas that were similar in tree density to each other.

### 2.3 Arthropod counts

Arthropod community composition and abundance were also sampled during the wet season of February 2022 using a circular sweep net with a diameter of 40 cm (Little et al., 2013; Nkwabi et al., 2011). We used sweep netting because it is a robust method to sample a wide range of arthropod taxa from a variety of habitats (Yi et al., 2012). For consistency, samples were collected by the same individual (KS) on warm, windless days. For each treatment plot, we conducted 100 sweeps per transect (a sweep is made with each stride), resulting in 300 sweeps per treatment. Each transect was separated by ~20 m. We repeated this sampling method for each treatment plot at each site. All arthropods were preserved in ethanol for identification to the morphospecies level (Oliver & Beattie, 1996)—a sufficient resolution to detect taxonomic responses to habitat management at local scales (Benton et al., 2002; Farrell et al., 2015). At the first instance of each morphospecies, the specimen was identified and labeled. This nomenclature was used consistently thereafter. All morphospecies were classified to the Family level by the same individual (TO) (see Table S1). Voucher specimens were preserved and retained.

### 2.4 Bird counts

Following Sirami et al. (2009), we estimated bird community composition and abundance during a single visit to our sites (February 25, 2022). We sampled during the austral summer when bird activity (including calling by breeding individuals) was at its peak (Hockey et al., 2006) improving individual bird detectability. The aim of our sampling was not to generate a full bird species inventory for our study area, nor was it an attempt to generate a longitudinal dataset post vegetation disturbance. All treatments were sampled between 08:30 and 14:00 on the day of sampling. Logistics prevented us from sampling directly after sunrise, which is generally considered to be the time of peak bird activity (Ralph et al., 1995). Nevertheless, a rarefaction analysis confirmed sampling completeness (Chao & Jost, 2012). Please see Figure S1 and the Appendix S1 for details regarding the rarefaction analysis. Because treatment plots varied in size, we accounted for this variation in our statistical analyses. Furthermore, the openness of the mowed treatment plots (Figure 1) ensured that the different plot sizes did not influence detectability (i.e., <100 m) (Sirami et al., 2009).

To conduct the bird observations, we used a two‐step approach. First, we used a double observer point count method within each treatment plot, using the edge of each plot as the detection cutoff. For this approach, one observer stood at a fixed point for 10 min recording all bird detections (visuals and calls) within the treatment plot while the second observer recorded and added any missed detections (Newman et al., 2018). As per Little et al. (2013), we only recorded birds that directly utilized the sampling area. Birds that were simply flying over and not interacting with the sampling area in any way were not counted. However, birds that were flying over and that did interact with/utilize the sampling area were included (Little et al., 2013). For example, a White‐backed Vulture (*Gyps africanus*) soaring several hundred meters above the plot would have been excluded, but a Barn Swallow (*Hirundo rustica*) hawking insects 1–2 m above the grass layer would have been included. The Spectacled Weaver (*Ploceus ocularis*), Village Weaver (*P. cucullatus*), Southern Masked Weaver (*P. velatus*), and Lesser Masked Weaver (*P. intermedius*) were treated as one morpho‐species (Weaver) due to the difficulty in distinguishing between the females whilst in a flock, and male birds that may have been in nonbreeding or eclipse plumage (Parker, 2019). Furthermore, all ground‐dwelling birds were excluded from the point counts. To account for ground‐dwelling birds, we used a second approach where we dragged a 30 m rope along an 80 or 100 m transect (transect size influenced by treatment size—but accounted for in statistical analyses below). In grass‐dominated habitats, this approach can be more accurate than point counts (Bibby, 2000) because it prevents birds from hiding in tall grass swards. Only ground‐dwelling birds (e.g., African Pipits, *Anthus cinnamomeus*; see Table 1) that were disturbed by the rope drags were recorded. We then combined records from both the point counts and rope drags to determine the bird community composition and abundance for each treatment plot. Due to the size of the treatment plots and to avoid double counting, we conducted a single point count and rope drag per treatment except for Site B summer and winter mow (3.6 and 4.6 ha, respectively) where replicate observations were made. These replicate observations were separated by at least 150 m to increase statistical independence of the observations (Naidoo, 2004; Parker, 2019). The same observers (KS and DP) performed the point counts and rope drags. Treatment plots were sampled randomly to reduce potential bias associated with time of day. We found that time of day/sequence of sampling did not influence bird detectability across treatments (Appendix S1 and Table S1; *χ* ^2^ = 6.26; df = 6; *p* = .40). Finally, no rain or noticeable changes in wind speeds were experienced on the day the birds were sampled.

**TABLE 1:** Bird species presence and their descriptions, following Hockey et al. (2006), across the different treatments.

Scientific name	Common name	Control	Rest	Summer mow	Winter mow	Primary food	Foraging strategy	Nesting category	Movement
*Crecopsis egregia*	African Crake					Carnivore	Ground	Ground	Seasonal
*Upupa africana*	African Hoopoe					Insectivore	Ground	Cavity	Resident
*Anthus cinnamomeus*	African Pipit					Insectivore	Ground	Ground	Resident
*Hirundo rustica*	Barn Swallow					Insectivore	Hawks	Non‐breeding	Seasonal
*Lybius torquatus*	Black‐collared Barbet					Frugivore	Frugivore	Cavity	Resident
*Oriolus larvatus*	Black‐headed Oriole					Insectivore	Gleans	Cup	Resident
*Uraeginthus angolensis*	Blue Waxbill					Granivore	Ground	Oval/ball	Resident
*Lamprotornis australis*	Burchell's Starling					Insectivore	Ground	Cavity	Resident
*Lamprotornis nitens*	Cape Glossy Starling					Frugivore	Frugivore	Cavity	Resident
*Streptopelia capicola*	Cape Turtle Dove					Granivore	Ground	Cup	Resident
*Batis molitor*	Chinspot Batis					Insectivore	Gleans	Cup	Resident
*Muscicapa adusta*	Dusky Flycatcher					Insectivore	P & S	Cup	Resident
*Mirafra rufocinnamomea*	Flappet Lark					Insectivore	Ground	Ground	Resident
*Dicrurus adsimilis*	Fork‐tailed Drongo					Insectivore	Hawks	Cup	Resident
*Emberiza flaviventris*	Golden‐breasted Bunting					Insectivore	Ground	Cup	Resident
*Passer diffusus*	Gray‐headed Sparrow					Granivore	Ground	Cavity	Resident
*Clamator levaillantii*	Levaillant's Cuckoo					Insectivore	Gleans	Brood parasite	Seasonal
*Coracias caudatus*	Lilac‐breasted Roller					Insectivore	P & S	Cavity	Resident
*Merops pusillus*	Little Bee‐eater					Insectivore	Hawks	Burrow	Resident
*Vidua paradisaea*	Paradise Whydah					Granivore	Ground	Brood parasite	Resident
*Vidua macroura*	Pin‐tailed Whydah					Granivore	Ground	Brood parasite	Resident
*Cisticola chiniana*	Rattling Cisticola					Insectivore	Gleans	Oval/ball	Resident
*Cecropis semirufa*	Red‐breasted Swallow					Insectivore	Hawks	Mud	Seasonal
*Rhinopomastus cyanomelas*	Scimitarbill					Insectivore	Bark	Cavity	Resident
*Melaniparus niger*	Southern Black Tit					Insectivore	Bark	Cavity	Resident
*Halcyon chelicuti*	Striped Kingfisher					Insectivore	P & S	Cavity	Resident
*Ploceidae*	Weaver					NA	NA	NA	NA
*Prionops plumatus*	White Helmetshrike					Insectivore	Gleans	Cup	Resident
*Crithagra mozambica*	Yellow‐fronted Canary					Granivore	Ground	Cup	Resident
*Cisticola juncidis*	Zitting Cisticola					Insectivore	Gleans	Oval/ball	Resident

*Note*: Presence data (gray blocks) indicate species turnover across treatments.

### 2.5 Data analysis

#### 2.5.1 Vegetation structure

We analyzed the vegetation responses to the different habitat management treatments using generalized linear mixed models from the *lme4* package in R (Bates et al., 2015). For all the below vegetation analyses, we included “Site” as a random factor. We analyzed the mean density of trees in each treatment using a Poisson distribution with a log link function. Because each treatment plot varied in size across the different sites, we included an offset term (log(size of each treatment plot)) within the model (Zuur, 2009), which resulted in an output of the number of trees per hectare. Treatment effects for both grass height and grass biomass were analyzed with Gamma distributions and log link functions. Finally, for variation in grass height, we used a binomial distribution because of its proportional nature. We then used a Sidak post hoc analysis to elucidate differences between treatments.

#### 2.5.2 Community composition

We assessed arthropod and bird species community compositions with nonmetric multidimensional scaling (nMDS) and Bray–Curtis dissimilarities using the *vegan* package in R (Oksanen et al., 2020). We tested for significant differences in community composition across treatments in the nMDS ordination using permutational multivariate analysis of variance (PERMANOVA). We ran separate PERMANOVAs for arthropod and bird communities. For these analyses, we used a nested (e.g., block) design so that permutations were constrained within the variable “Site.” Because insect sampling was conducted over standardized areas, we used absolute counts in the PERMANOVA; however, for birds, we used relative abundances because sampled areas differed in size. Finally, we used the *envfit* function to plot species vectors on the ordination plots to identify the species driving the observed differences between treatments. For birds, we set *α* = .05; however, for arthropods, we set *α* = .01 to limit the number of morphospecies displayed (*n* = 4) on the ordination plots.

#### 2.5.3 Patterns of species richness and abundance

We analyzed differences in arthropod richness and abundance using separate generalized linear mixed models. Both models included “Site” as a random factor and had Poisson distributions with log link functions. For these models, we did not need to include an offset variable for size because the same area was sampled at each treatment plot and each site (i.e., 300 sweeps per treatment with the same sized sweep net). We also used generalized linear mixed models to analyze treatment effects on bird richness and abundance. As above, these models included “Site” as a random factor and had Poisson distributions with log link functions. However, because the size of the treatment plots varied (see above), which influences species–area relationships (MacArthur & Wilson, 2001), we included an offset variable (log(size of each treatment plot)) within the model (Zuur, 2009), resulting in an output of bird richness per hectare and bird abundance per hectare, respectively.

We found that treatment significantly influenced the richness and abundance of both arthropods and birds (see below). Consequently, we assessed whether environmental variables (i.e., vegetation metrics above) and/or the abundance of their prey (i.e., arthropods for insectivorous birds) or predators (i.e., insectivorous birds for arthropods) influenced our observed patterns. For this analysis, we were only interested in insectivorous birds, which were the dominant feeding guild in our study (see below). To determine what factors may influence insect abundance and richness, we modeled mean arthropod abundance and richness (dependent variables in two separate models) against the following independent variables: average grass height, average grass biomass, variation in grass height, woody plant density, and the abundance of insectivorous birds. For these analyses, we used a generalized linear mixed model with a gamma distribution, log link function, and included “Site” as a random factor. We repeated the above process and modeled, in separate models, the abundance, and richness of insectivorous birds (dependent variable) against the following variables: average grass height, average grass biomass, variation in grass height, woody plant density, and the abundance of arthropods. For these analyses, we used a generalized linear mixed model with a poisson distribution, log link function, and included “Site” as a random factor. Furthermore, for all models, we tested for multicollinearity and automated the model selection process using the *dredge* function from the *MuMIn* package (Barton, 2020). All co‐linear models that had a correlation coefficient of >0.6 between predictor variables were removed and the best fit model was determined using AICc and Akaike weights (Burnham & Anderson, 2010).

## 3 RESULTS

### 3.1 Vegetation structure

**FIGURE 2:**
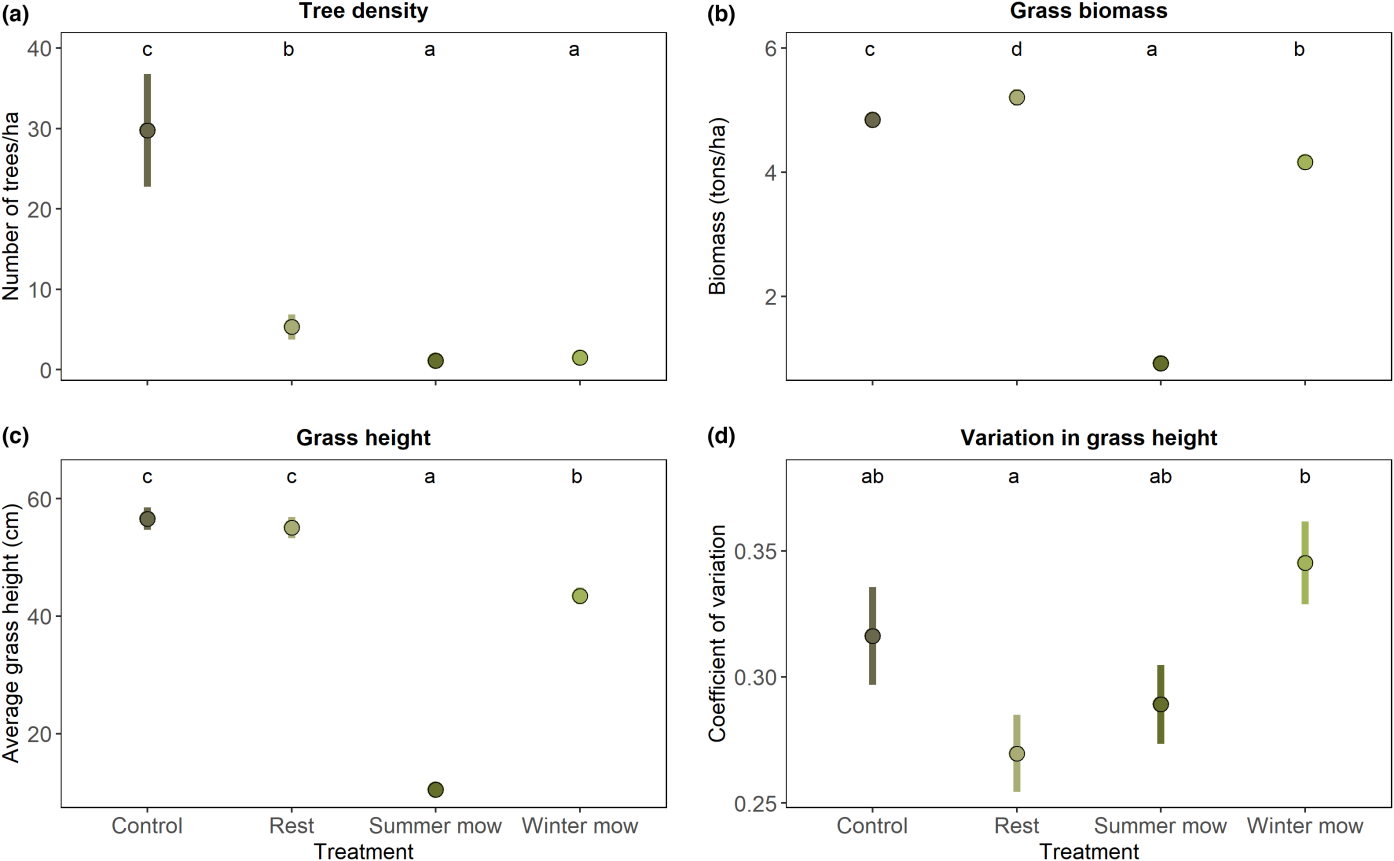
Vegetation metrics across treatments (mean ± SE). Panel (a) shows tree density, (b) grass biomass, (c) grass height, and (d) variation in grass height. Means with no letter in common are significantly different (*α* = .05).

The four treatments significantly influenced tree density (*χ* ^2^ = 157.81, df = 3, *p* < .001), grass biomass (*χ* ^2^ = 3597.7, df = 3, *p* < .001), average grass height (*χ* ^2^ = 4663.4, df = 3, *p* < .001), and the variation in grass height (*χ* ^2^ = 13.45, df = 3, *p* = .004) (Figure 2). Control areas had the highest tree density (~30 trees/ha) of all treatments, followed by rest plots that had a significantly lower tree density than the control areas (~5 trees/ha), but higher than both the summer and winter mow plots. The summer mow and winter mow plots had the lowest densities with both plots having similar tree densities (~1 trees/ha) (Figure 2a). Grass biomass was significantly different across all treatments with the highest biomass occurring in rest plots (5.2 tons/ha), followed by control areas (4.8 tons/ha), winter mow plots (4 tons/ha), and finally, the summer mow plots (1 ton/ha) (Figure 2b). With respect to grass height, control areas and rest plots had the highest grass height, which was similar (~58 and ~55 cm, respectively), followed by winter mow plots (~43 cm), and then summer mow plots (~10 cm) (Figure 2c). For variation in grass height, we only observed significant difference between rest and winter mow plots. Winter mow plots has the highest variation in grass height (~32%) with rest plots having the lowest (~27%) (Figure 2d).

### 3.2 Treatment effects on community composition, species richness, and abundance

#### 3.2.1 Arthropods

**FIGURE 3:**
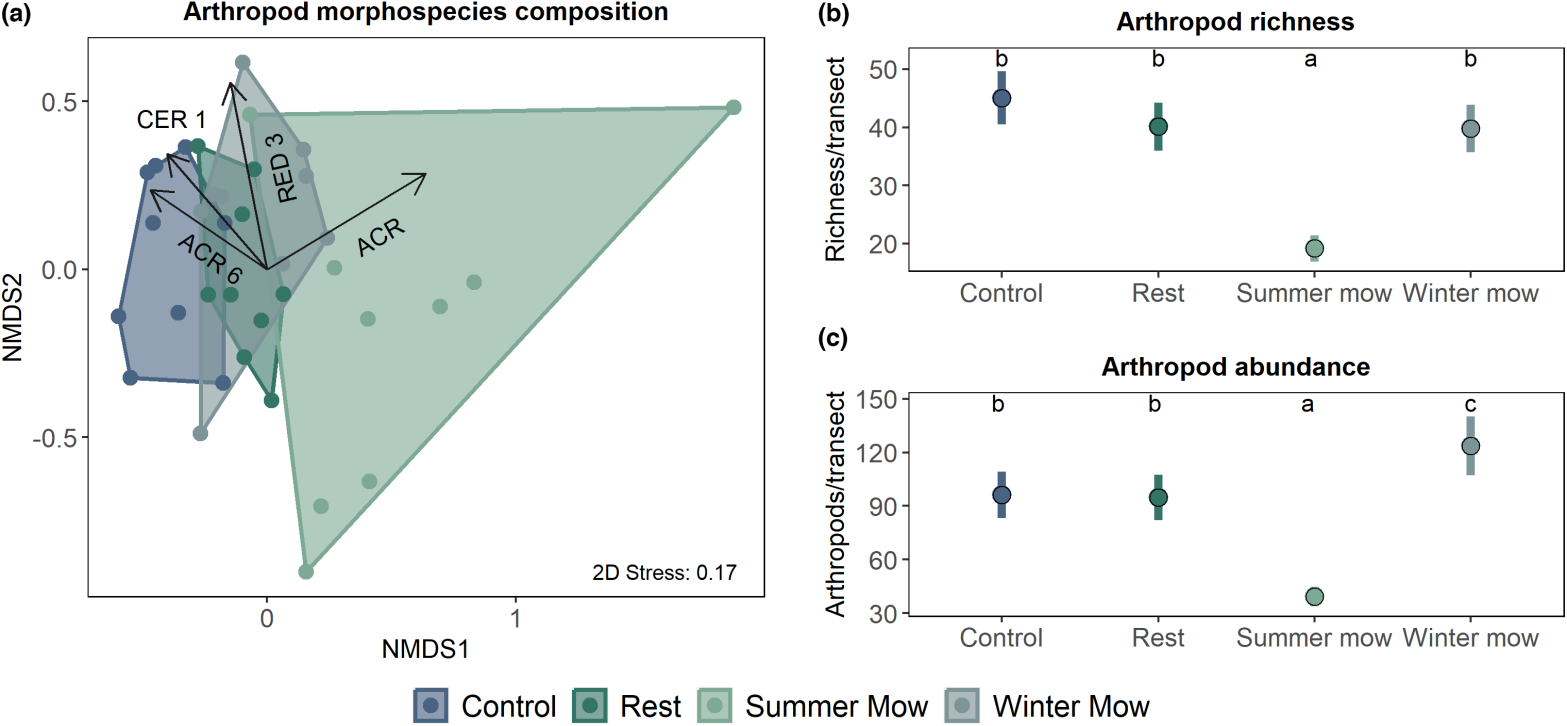
Treatment effects on arthropods. Panel (a) illustrates the differences in community composition of arthropod morphospecies across treatments. The arrows are species vectors, which represent the species significantly associated with each treatment (*α* = .01). Panel (b) shows patterns of arthropod richness (mean ± SE) across treatments. Panel (c) depicts patterns of arthropod abundance across treatments. For each panel (b) and (c), means with no letter in common are significantly different (*α* = .05).

We collected a total of 3269 arthropods from 15 orders that were subsequently identified to 179 morphospecies (Table S2). Of the 15 orders, five made up ~87% of all collected arthropods: Araneae: ~34.5%, Hemiptera: ~19.5%, Coleoptera: ~14.5%, Orthoptera: ~13%, and Hymenoptera: ~5.5% (Figure 3). Arthropod community composition was significantly influenced by treatments (PERMANOVA pseudo*F*_3,35_ = 2.89, *p* = .001, Figure 3a). Of the 179 morphospecies, four morphospecies were characteristic of specific treatments at a confidence interval of 99% (i.e., *α* = .01). Morphospecies “ACR 6” (Order: Orthoptera, Suborder: Caelifera, Family: Acrididae) is a small grasshopper and was characteristic of control areas, “CER 1” (Order: Hemiptera, Suborder: Auchenorrhyncha, Family: Cercopidae) is a sap sucker that is indicative of rest and control treatments, “RED 3” (Order: Hemiptera, Suborder: Heteroptera, Family: Reduviidae) is a small assassin bug, which was associated with winter mow and rest areas, while “ACR” (Order: Orthoptera, Suborder: Caelifera, Family: Acrididae) is another grasshopper, which was associated with summer mows (Figure 3a).

We found that arthropod richness and abundance were significantly influenced by treatment (arthropod morphospecies richness: *χ* ^2^ = 113.8, df = 3, *p* < .001, arthropod abundance: *χ* ^2^ = 444.7, df = 3, *p* < .001, Figure 3b,c) with both these metrics responding similarly across the different treatments. Control areas, rest plots, and winter mow plots had nonsignificantly different richness of arthropods (~40 morphospecies/transect). By contrast, summer mow plots had significantly lower arthropod richness than the other treatments (~15 morphospecies/transect). Winter mow plots had significantly higher arthropod abundance (~120 arthropods/transect) than other treatments (Figure 3c). Both control areas and rest plots had similar abundances of arthropods (~90 arthropods/transect), which were significantly lower than the winter mow treatment, but significantly higher than summer mow plots, which had the lowest abundance of arthropods (~35 arthropods/transect). Our model selection process identified herbaceous biomass as the best predictor of arthropod richness and abundance (Tables S3 and S4). Specifically, we found that grass biomass positively influenced both arthropod abundance (*t* = 4.43, *p* < .001, *R* ^2^ = .67) and arthropod richness (*t* = 6.97, *p* < .001, *R* ^2^ = .83).

#### 3.2.2 Birds

**FIGURE 4:**
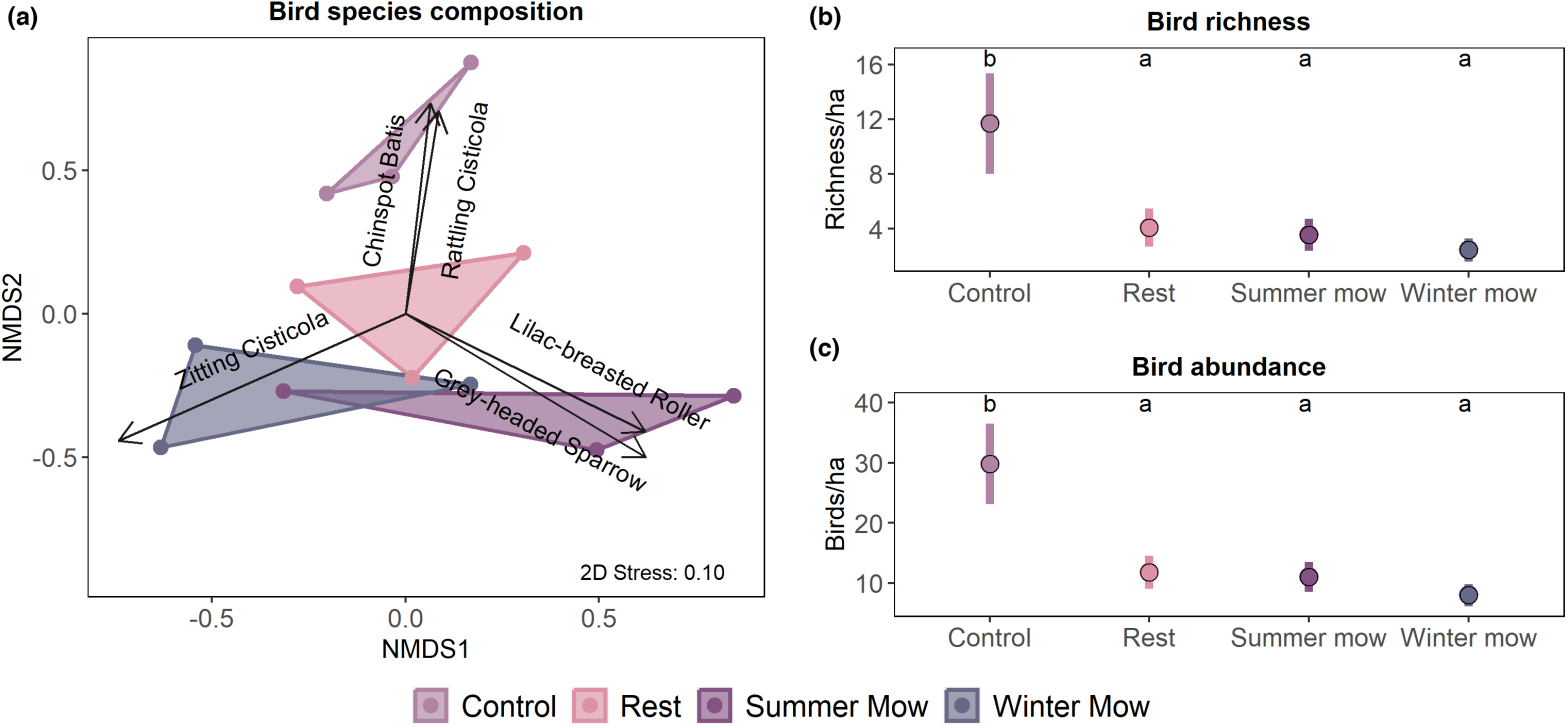
Treatment effects on birds. Panel (a) illustrates the differences in community composition of bird species across treatments. The arrows are species vectors, which represent the species significantly associated with each treatment (*α* = .05). Panel (b) shows patterns of bird species richness (mean ± SE) across treatments. Panel (c) depicts patterns of bird abundance across treatments. For each panel (b) and (c), means with no letter in common are significantly different (*α* = .05).

We observed a total of 222 individual birds from 30 species, of which ~80% were insectivores, ~17% were granivores, ~2% were frugivores, and <1% were carnivores (Table 1). Like the arthropods, bird community composition was significantly different across treatments (PERMANOVA pseudo*F*_3,11_ = 1.94, *p* = .026, Figure 4a). Five bird species were significantly associated with the differences in community composition among treatments. Lilac‐breasted Rollers (*Coracias caudatus*) and Gray‐headed Sparrows (*Passer diffuses*) were associated with the summer mow treatments, Zitting Cisticolas (*Cisticola juncidis*) were associated with the winter mow treatments, and Chinspot Batis' (*Batis molitor*) and Rattling Cisticolas (*Cisticola chiniana*) were associated with the control plots. Of these five bird species, four are classified as insectivores.

We found that bird richness and abundance were significantly influenced by treatment (bird species richness: *χ* ^2^ = 24.1, df = 3, *p* < .001, bird abundance: *χ* ^2^ = 47.8, df = 3, *p* < .001; Figure 4b,c) with both these metrics responding similarly across the different treatments. Control areas had significantly higher richness (~12 species/ha) and abundance of birds (~30 birds/ha) than the other treatments (Figure 4b,c). Rest, summer, and winter mow plots had comparable bird richness (~4 species/ha) and bird abundance (~10 birds/ha), respectively. With the observed differences in bird richness across treatments, we detected species turnover across treatments, with control areas having eight unique species, rest plots two unique species, summer mows plots five unique species, and winter mow plots two unique species (Table 1).

When we analyzed the response of only insectivorous birds across treatments, we found the same response compared to when all birds were analyzed together (above). Again, control areas had significantly more insectivorous birds (~25 birds/ha) compared with the other treatments, which all had similar abundance of insectivorous birds (~9 birds/ha; Figure S3). Our model selection process identified tree density as the best predictor of insectivore bird richness and abundance (Tables S5 and S6). Specifically, we found that tree density positively influenced both insectivore bird abundance (*z* = 7.12, *p* < .001, *R* ^2^ = .79) and the richness of insectivorous birds (*z* = 5.41, *p* < .001, *R* ^2^ = .67).

## 4 DISCUSSION

The management‐induced changes in vegetation structure observed in our study resulted in significant differences in both arthropod and bird communities. Specifically, the three treatments with higher grass biomass supported greater arthropod richness and abundance. By contrast, the plots that had the highest tree density supported greater bird richness and abundance. Habitat structure and herbaceous cover are established drivers of animal communities (Aranda & Graciolli, 2015; Brüggeshemke et al., 2022; Prather & Kaspari, 2019; Schmitt et al., 2022), as such, any habitat management practice that affects vegetation structure and cover will also impact the animals relying on the vegetation. However, we show that these impacts are variable depending on the taxonomic group being assessed.

We found that the biomass of the herbaceous layer was a key driver of arthropod abundance and richness. Similarly, Nkwabi et al. (2011) reported that arthropod abundance was highest in undisturbed grassland habitats in the Serengeti, Tanzania. Furthermore, Little et al. (2013) demonstrated that unburnt (taller) grasslands supported higher arthropod biomass than burnt (shorter) patches. The two arthropod orders (i.e., Araneae and Hemiptera) that contributed approximately half of all the arthropods that we collected across our sites have been found to respond strongly to vegetation structure. These patterns are likely driven by the relationship between vegetation structure and predator prey‐dynamics within arthropods (Prather & Kaspari, 2019). For example, high biomass provides suitable cover for predatory species (e.g., Araneae—spiders) (Warui et al., 2005) and refugia for nonpredatory species (e.g., Hemiptera—sapsuckers) (Prather & Kaspari, 2019). Crucially, because our manipulated sites were mowed at different times of the year (i.e., winter and summer), the herbaceous biomass was higher in the winter mow treatments than the summer mow treatments. By having mow treatments that are applied at different times of the year, we could tease apart the importance of herbaceous layer biomass, rather than trees, in driving arthropod communities. While mowing had the same effect on tree density (i.e., very low), the reduced herbaceous biomass available at the summer mow sites negatively affected arthropod abundance and richness, while winter mow plots that had higher herbaceous biomass hosted high abundance and richness of arthropods.

Heterogeneity within habitats is also an important driver of diversity and abundance of bird communities. In other systems, both bird diversity and abundance generally increase in habitats that have more complex vertical and horizontal architectures because a greater variety of resources are available and there is greater potential for segregation at the microhabitat/niche level (Brüggeshemke et al., 2022; MacArthur & MacArthur, 1961; Martin & Possingham, 2005; Parker, 2019). This relationship may explain the observed patterns of high bird abundance and richness in our control plots than the other treatment plots. Furthermore, Seymour and Dean (2009) and Ogada et al. (2008) demonstrated that insectivorous birds that tend to perch and sally to catch insect prey are more commonly associated with habitats that have large trees present (i.e., our control plots). Our findings support these results in that all the insectivorous bird species that we observed were perch and sally hunters (Hockey et al., 2006) and these birds dominated the control plots. The higher density of trees in our control plots likely provided important perching stations for these species. It is possible, however, that the higher visibility in the plots that had lower grass biomass (i.e., the summer mow) also enhanced insect prey catchability, similar to what has been found in other South African systems (Musgrave & Compton, 1997). This enhanced catchability could explain why Lilac‐breasted Rollers characterized summer mow habitats where they fed on potentially easy‐to‐detect arthropods due to low herbaceous vegetation cover (K. Stears, personal observations). Although interactive effects have been observed between the *prey abundance* and *prey catchability* hypotheses for some predators (Smith et al., 2020), numerous predators, including insectivorous birds, prefer to hunt in habitats where it is easier to catch their prey and not necessarily where prey are more abundant (Kämpfer et al., 2022; Kämpfer & Fartmann, 2019; Nkwabi et al., 2011). Our results support the *prey catchability* hypothesis because tree density (i.e., perching locations for hunting), and not arthropod abundance, was the overriding driver of insectivorous bird abundance and richness across our treatments.

Similar to Parker (2019), we show disparate responses among insectivorous birds in the face of disturbances that alter vegetation structure (see also Banks et al., 2017; Ehlers Smith et al., 2015). Consequently, we observed bird species turnover across our treatments (Table 1). For example, the African Hoopoe (*Upupa africana*), which prefers short grassland for foraging (Hockey et al., 2006), was only found in summer mow treatments. By contrast, the Striped Kingfisher (*Halcyon chelicuti*), a perch and sally specialist (Hockey et al., 2006), was only found in the control areas. Thus, at the landscape scale, the different mow treatments add to habitat heterogeneity and increase species richness for birds (MacArthur & Wilson, 2001). This finding underscores the important relationship between habitat heterogeneity and species richness. However, if a given management approach was to become widespread, this would likely counteract the habitat heterogeneity paradigm because of the reduction in habitat heterogeneity and the subsequent reduction in species richness (see also Schmitt et al., 2022; Zhou et al., 2021).

Our rarefaction analysis indicated that we achieved sampling completeness (Appendix S1 and Figure S1). However, we recognize that some bird species may have been missed during sampling due to the inherent variability in bird detectability when using point counts (Brotons et al., 2005; Butler et al., 2005). With respect to arthropods, sweep netting does not necessarily detect all arthropods that may be present (Blaum et al., 2009). Moreover, sweep netting does not sample arthropods that reside on and within woody vegetation. However, if we were to sample arthropods from woody vegetation, these data would likely strengthen our observed patterns due to the near lack of woody vegetation in all mow treatments. Finally, our mow treatment plots within a site are all adjacent to each other (i.e., the treatment line is shared between plots), which could potentially lead to spillover in use by vagile species across treatment plots. However, we found clear patterns of use across treatments with respect to species composition.

Ultimately, we have shown experimentally that understanding the comparative effects of habitat management interventions on arthropod (prey) and bird (predator) communities can elucidate the ecological consequences of such management approaches. Given the burgeoning need to implement vegetation management practices to combat increasing woody plant cover, our results demonstrate the importance of understanding the outcomes of a similar management approach (i.e., mowing) that is applied at different temporal scales (i.e., annual summer mow, annual winter mow, >5‐year gap since last mow). Disturbances that influence consumer–resource interactions can shape individual behavior, populations, and define ecosystem structure and functioning (Karakoç et al., 2018; Stears & McCauley, 2018). African savannas are the summer (i.e., wet season) feeding and breeding grounds for a wide range of migratory bird species (and provide the habitat for their prey: arthropods). Thus, it is imperative to be cognizant of the impacts that management practices that impact wet season vegetation have on the behaviors of both resident and migratory birds.

## AUTHOR CONTRIBUTIONS

**Dan M. Parker:** Conceptualization (equal); data curation (equal); formal analysis (equal); funding acquisition (equal); investigation (equal); methodology (equal); project administration (supporting); resources (lead); supervision (lead); validation (equal); visualization (supporting); writing – original draft (lead); writing – review and editing (equal). **Keenan Stears:** Conceptualization (equal); data curation (equal); formal analysis (equal); funding acquisition (equal); investigation (equal); methodology (equal); project administration (equal); validation (equal); visualization (lead); writing – original draft (supporting); writing – review and editing (equal). **Terence Olckers:** Data curation (equal); formal analysis (equal); validation (equal); writing – review and editing (equal). **Melissa H. Schmitt:** Conceptualization (equal); data curation (equal); formal analysis (equal); funding acquisition (equal); investigation (equal); methodology (equal); project administration (equal); validation (equal); visualization (supporting); writing – original draft (supporting); writing – review and editing (equal).

## CONFLICT OF INTEREST STATEMENT

The authors declare no conflict of interests.

## Supporting information


Appendix S1

## Data Availability

All data and code can be found on the Zenodo repository (Parker et al., 2022).
