# Supplementary material for: Vegetation management shapes arthropod and bird communities in an African savanna
Source: Ecol Evol. 2023 Mar 8;13(3):e9880. doi: 10.1002/ece3.9880 (PMC9994611; doi:10.1002/ece3.9880)
Supplement: Supplementary file 1 — Appendix S1 [file ECE3-13-e9880-s001.docx]

**Supplementary Material**

**Vegetation management shapes arthropod and bird communities in an African savanna**

Testing for sampling completeness:

We assessed sampling completeness for the bird sampling using a rarefaction procedure for each treatment (Colwell & Coddington 1994). To do this, we plotted the observed species richness, interpolated using the Mao Tau method, against the Chao 1 Abundance-based Coverage Estimator (ACE) and the Chao 2 Incidence-based Coverage Estimator (ICE) following Colwell & Coddington (1994). Sampling was deemed complete when the 95 % confidence intervals for the observed (Mao Tau) and estimated (Chao 1 and Chao 2) values overlapped (sensu Robinson *et al.* 2007). The interpolated species richness and Chao 1 ACE and Chao 2 ICE values for each treatment are shown in Fig. S1. Because the 95% confidence intervals (error bars) of the observed species richness (Mao Tau) and the two estimators overlap for each treatment after replicate three, sampling for each treatment was deemed adequate (Fig. S1) (Robinson *et al.* 2007).


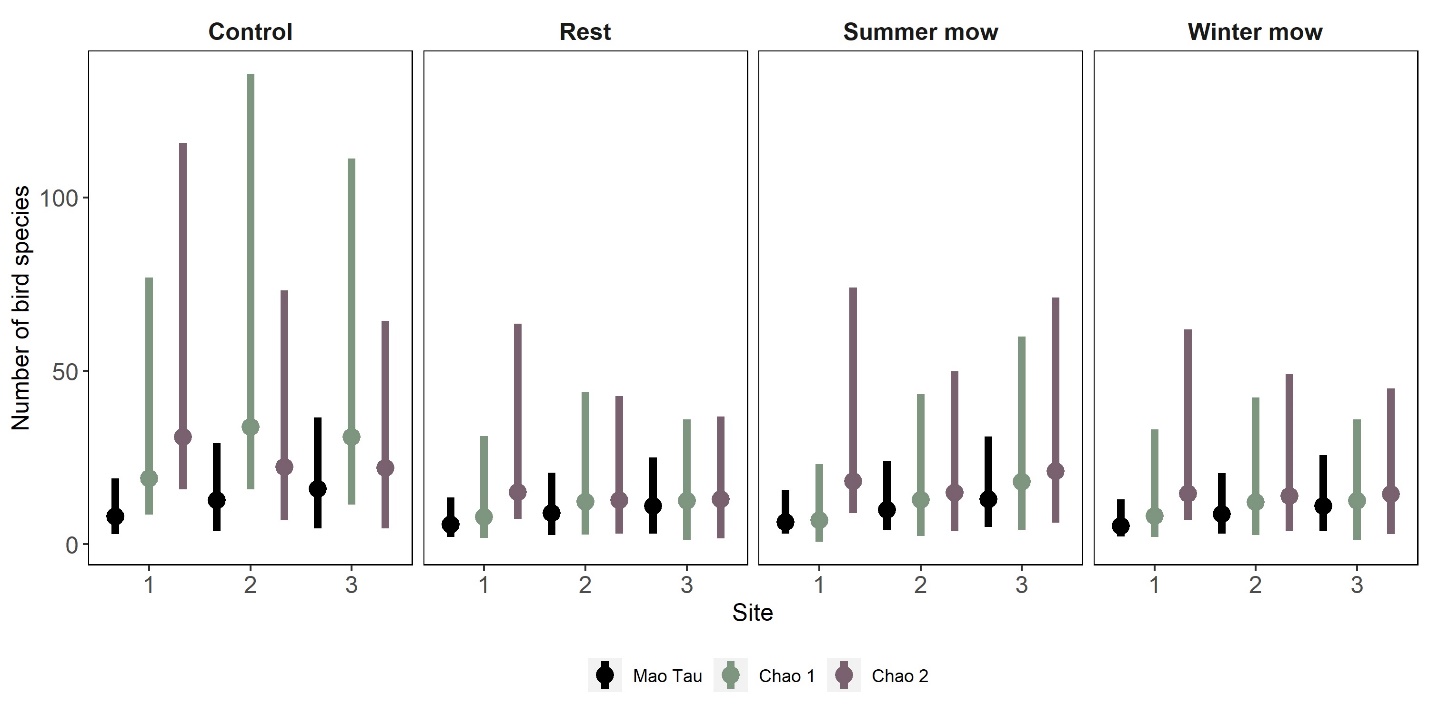


Fig S1: The observed bird species richness (Sobs Mao Tau - black), the Chao 1 Abundance-based Coverage Estimator (green), and the Chao 2 Incidence-based Coverage Estimator (purple) for each replicate within each treatment (i.e., control, rest, summer mow and winter mow) at MalaMala Game Reserve, South Africa. Error bars represent 95 % confidence intervals.


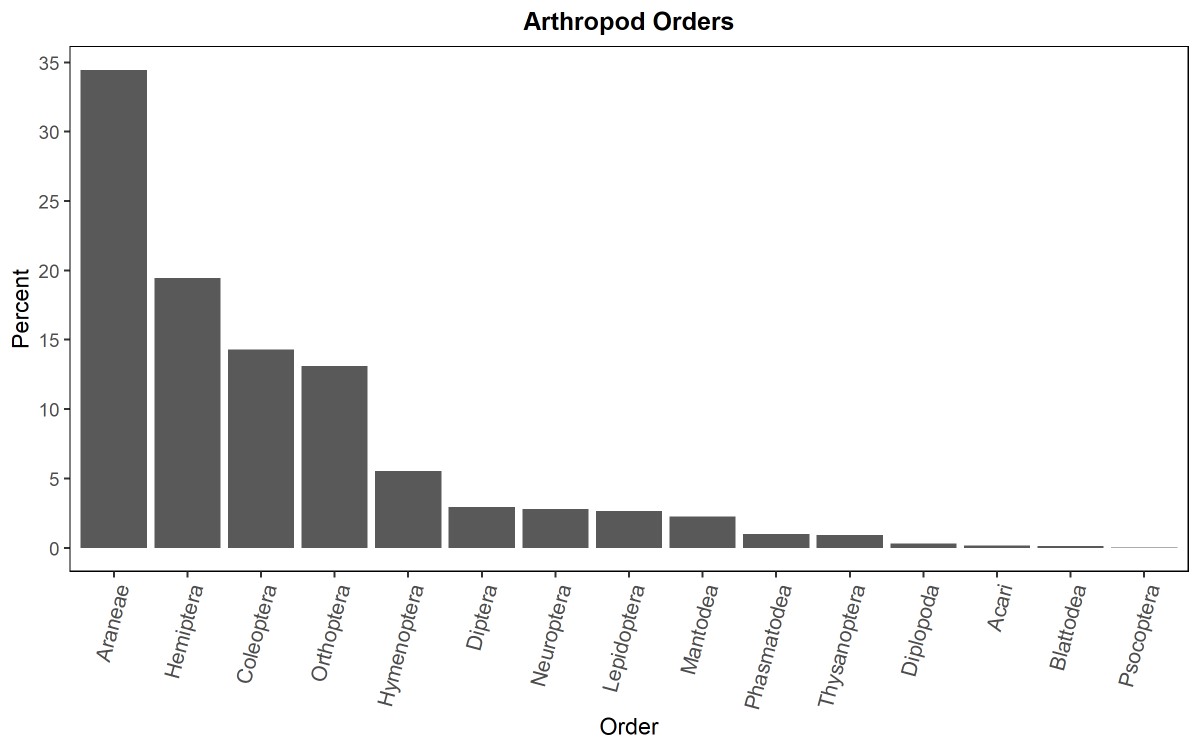


Fig S2: Percent contribution of arthropod orders collected across all treatments.


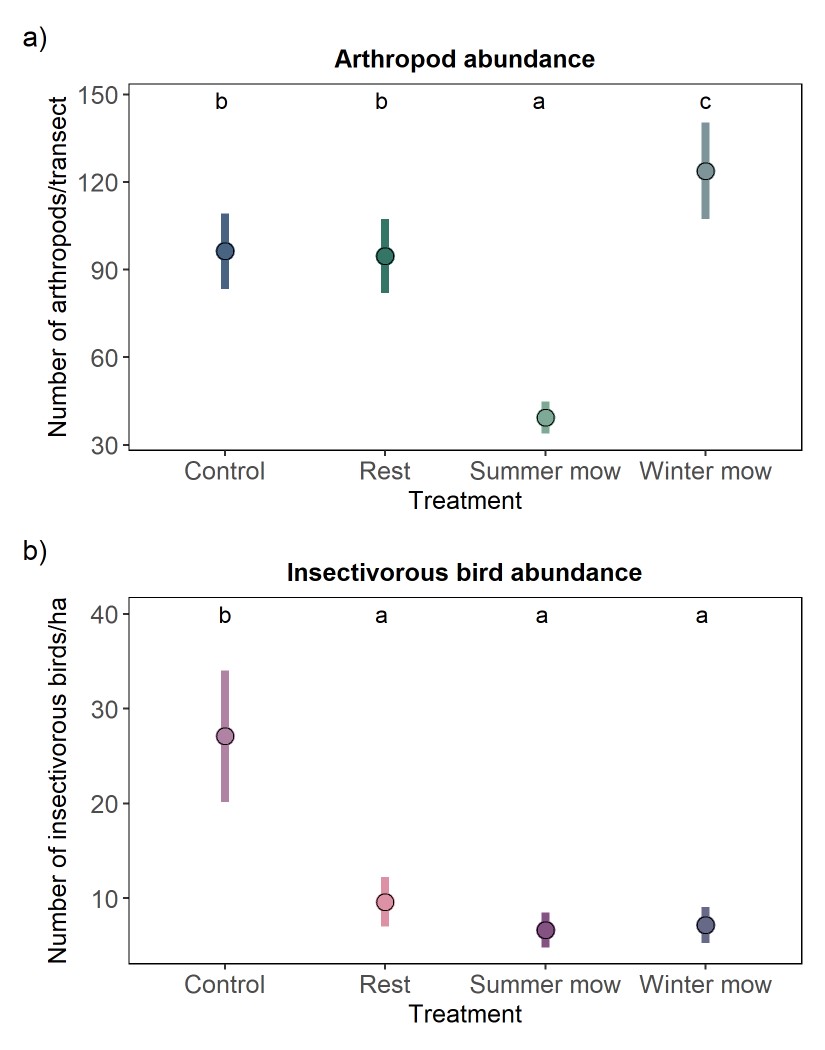


Fig S3: Insectivorous bird abundance (mean + SE) across treatments. Means with no letter in common are significantly different (α = 0.05).

Testing for the effect of time of day on bird sampling:

The detectability of birds is known to be influenced by the time of day, with detectability generally declining after the first few hours following sunrise (Ralph *et al.* 1995). Due to logistical constraints, we were only able to access the site to conduct our bird sampling after 08:30. This restriction precluded us from conducting our bird sampling during what could be considered the ‘best’ time to detect as many species as possible (Bibby 2000; Ralph *et al.* 1995). As such, it was important that we tested whether time of day had any effect on our bird sampling. We conducted a Chi-square test of independence on the number of species observed across the treatments. The raw data for this test, along with the approximate time that each replicate was sampled are provided in Table S1. The Chi-square test of independence was run in R (R Core Team 2021). There was no significant association between when a replicate within a treatment was sampled (i.e., time of day had no significant effect on our sampling; χ2 = 6.26; df = 6; P = 0.40).

Table S1**:** The observed number of bird species and approximate time that each replicate was sampled for each treatment at MalaMala Game Reserve, South Africa in February 2022.

| **Treatment** | **Replicate 1**  **(time) [# species]** | **Replicate 2**  **(time) [# species]** | **Replicate 3**  **(time) [# species]** |
| --- | --- | --- | --- |
| Control | (11:00) [8] | (11:30) [4] | (13:30) [12] |
| Rest | (10:00) [7] | (09:00) [5] | (13:00) [5] |
| Winter mow | (11:00) [4] | (09:30) [6] | (12:30) [6] |
| Summer mow | (10:30) [4] | (08:30) [9] | (12:00) [6] |

Table S2: List of arthropod morphospecies (sorted by Order) caught across our treatments and their descriptions, including their taxonomy, and feeding strategies. Identifications were made following Picker *et al.* (2019).

| **Morphospecies** | **Order** | **Suborder** | **Family** | **Guild** |
| --- | --- | --- | --- | --- |
| ACA | Acari |  | Ixodidae | Parasite |
| ARA | Araneae |  | Unknown | Predator |
| ARA 1 | Araneae |  | Unknown | Predator |
| ARA 10 | Araneae |  | Unknown | Predator |
| ARA 11 | Araneae |  | Unknown | Predator |
| ARA 12 | Araneae |  | Unknown | Predator |
| ARA 13 | Araneae |  | Unknown | Predator |
| ARA 14 | Araneae |  | Unknown | Predator |
| ARA 2 | Araneae |  | Unknown | Predator |
| ARA 3 | Araneae |  | Unknown | Predator |
| ARA 4 | Araneae |  | Unknown | Predator |
| ARA 5 | Araneae |  | Unknown | Predator |
| ARA 6 | Araneae |  | Unknown | Predator |
| ARA 7 | Araneae |  | Unknown | Predator |
| ARA 8 | Araneae |  | Unknown | Predator |
| ARA 9 | Araneae |  | Unknown | Predator |
| BLA | Blattodea |  | Blattidae | Saprophage |
| BRE | Coleoptera | Polyphaga | Brentidae | Leaf feeder |
| BUP | Coleoptera | Polyphaga | Buprestidae | Leaf feeder |
| CAR | Coleoptera | Adephaga | Carabidae | Predator |
| CHR | Coleoptera | Polyphaga | Chrysomelidae | Leaf feeder |
| CHR 1 | Coleoptera | Polyphaga | Chrysomelidae | Leaf feeder |
| CHR 2 | Coleoptera | Polyphaga | Chrysomelidae | Leaf feeder |
| CHR 3 | Coleoptera | Polyphaga | Chrysomelidae | Leaf feeder |
| CHR 4 | Coleoptera | Polyphaga | Chrysomelidae | Leaf feeder |
| COC | Coleoptera | Polyphaga | Coccinellidae | Predator |
| COC 1 | Coleoptera | Polyphaga | Coccinellidae | Predator |
| COC 2 | Coleoptera | Polyphaga | Coccinellidae | Predator |
| COL | Coleoptera | Polyphaga | Unknown | Unknown |
| COL 1 | Coleoptera | Polyphaga | Unknown | Unknown |
| COL 2 | Coleoptera | Polyphaga | Unknown | Unknown |
| COL 3 | Coleoptera | Polyphaga | Unknown | Unknown |
| COL 4 | Coleoptera | Polyphaga | Tenebrionidae | Unknown |
| COL 5 | Coleoptera | Polyphaga | Unknown | Unknown |
| CUR | Coleoptera | Polyphaga | Curculionidae | Leaf feeder |
| CUR 1 | Coleoptera | Polyphaga | Curculionidae | Leaf feeder |
| CUR 2 | Coleoptera | Polyphaga | Curculionidae | Leaf feeder |
| CUR 3 | Coleoptera | Polyphaga | Curculionidae | Leaf feeder |
| CUR 4 | Coleoptera | Polyphaga | Curculionidae | Leaf feeder |
| CUR 5 | Coleoptera | Polyphaga | Curculionidae | Leaf feeder |
| DER | Coleoptera | Polyphaga | Dermestidae | Saprophage |
| ELA | Coleoptera | Polyphaga | Elateridae | Saprophage |
| LYC | Coleoptera | Polyphaga | Lycidae | Nectar feeder |
| MEL | Coleoptera | Polyphaga | Meloidae | Leaf feeder |
| SCA | Coleoptera | Polyphaga | Scarabaeidae | Saprophage |
| SCA 1 | Coleoptera | Polyphaga | Scarabaeidae | Saprophage |
| SCA 2 | Coleoptera | Polyphaga | Scarabaeidae | Saprophage |
| TEN | Coleoptera | Polyphaga | Tenebrionidae | Saprophage |
| MIL | Diplopoda |  | Unknown | Saprophage |
| MIL 1 | Diplopoda |  | Unknown | Saprophage |
| MIL 2 | Diplopoda |  | Unknown | Saprophage |
| ASI | Diptera | Brachycera | Asilidae | Predator |
| ASI 1 | Diptera | Brachycera | Asilidae | Predator |
| ASI 2 | Diptera | Brachycera | Asilidae | Predator |
| BOM | Diptera | Brachycera | Bombyliidae | Nectar feeder |
| CHI | Diptera | Nematocera | Chironomidae | Nectar feeder |
| DIO | Diptera | Brachycera | Diopsidae | Nectar feeder |
| DIP | Diptera | Brachycera | Muscidae | Saprophage |
| DIP 1 | Diptera | Brachycera | Muscidae | Saprophage |
| DIP 2 | Diptera | Brachycera | Muscidae | Unknown |
| DIP 3 | Diptera | Brachycera | Muscidae | Saprophage |
| EMP | Diptera | Brachycera | Empididae | Nectar feeder |
| SCI | Diptera | Nematocera | Sciaridae | Saprophage |
| SYR | Diptera | Brachycera | Syrphidae | Nectar feeder |
| TEP | Diptera | Brachycera | Tephritidae | Nectar feeder |
| TIP | Diptera | Nematocera | Tipulidae | Nectar feeder |
| CDA | Hemiptera | Auchenorrhyncha | Cicadidae | Sap sucker |
| CER | Hemiptera | Auchenorrhyncha | Cercopidae | Sap sucker |
| CER 1 | Hemiptera | Auchenorrhyncha | Cercopidae | Sap sucker |
| CER 2 | Hemiptera | Auchenorrhyncha | Cercopidae | Sap sucker |
| CER 3 | Hemiptera | Auchenorrhyncha | Cercopidae | Sap sucker |
| CIC | Hemiptera | Auchenorrhyncha | Cicadellidae | Sap sucker |
| CIC 1 | Hemiptera | Auchenorrhyncha | Cicadellidae | Sap sucker |
| CIC 2 | Hemiptera | Auchenorrhyncha | Cicadellidae | Sap sucker |
| CID | Hemiptera | Auchenorrhyncha | Cicadidae | Sap sucker |
| COR | Hemiptera | Heteroptera | Coreidae | Sap sucker |
| COR 1 | Hemiptera | Heteroptera | Coreidae | Sap sucker |
| COR 2 | Hemiptera | Heteroptera | Coreidae | Sap sucker |
| COR 3 | Hemiptera | Heteroptera | Coreidae | Sap sucker |
| COR 4 | Hemiptera | Heteroptera | Coreidae | Sap sucker |
| DEL | Hemiptera | Auchenorrhyncha | Delphacidae | Sap sucker |
| FUL | Hemiptera | Auchenorrhyncha | Fulgoridae | Sap sucker |
| HEM | Hemiptera | Auchenorrhyncha | Unknown | Sap sucker |
| HEM 1 | Hemiptera | Heteroptera | Unknown | Sap sucker |
| HEM 2 | Hemiptera | Heteroptera | Lygaeidae | Sap sucker |
| HEM 3 | Hemiptera | Auchenorrhyncha | Unknown | Sap sucker |
| HEM 4 | Hemiptera | Heteroptera | Unknown | Sap sucker |
| LYG 1 | Hemiptera | Heteroptera | Lygaeidae | Sap sucker |
| LYG 2 | Hemiptera | Heteroptera | Lygaeidae | Sap sucker |
| MAR | Hemiptera | Sternorrhyncha | Margarodidae | Sap sucker |
| MIR | Hemiptera | Heteroptera | Miridae | Predator |
| PEN | Hemiptera | Heteroptera | Pentatomidae | Sap sucker |
| PEN 1 | Hemiptera | Heteroptera | Pentatomidae | Sap sucker |
| PEN 2 | Hemiptera | Heteroptera | Pentatomidae | Sap sucker |
| PEN 3 | Hemiptera | Heteroptera | Pentatomidae | Sap sucker |
| PEN 4 | Hemiptera | Heteroptera | Pentatomidae | Sap sucker |
| PSL | Hemiptera | Sternorrhyncha | Psyllidae | Sap sucker |
| RED | Hemiptera | Heteroptera | Reduviidae | Predator |
| RED 1 | Hemiptera | Heteroptera | Reduviidae | Predator |
| RED 2 | Hemiptera | Heteroptera | Reduviidae | Predator |
| RED 3 | Hemiptera | Heteroptera | Reduviidae | Predator |
| RED 4 | Hemiptera | Heteroptera | Reduviidae | Predator |
| RED 5 | Hemiptera | Heteroptera | Reduviidae | Predator |
| RED 6 | Hemiptera | Heteroptera | Reduviidae | Predator |
| RHO | Hemiptera | Heteroptera | Rhopalidae | Sap sucker |
| SCU | Hemiptera | Heteroptera | Scutelleridae | Sap sucker |
| SCU 1 | Hemiptera | Heteroptera | Scutelleridae | Sap sucker |
| SCU 2 | Hemiptera | Heteroptera | Scutelleridae | Sap sucker |
| TEG | Hemiptera | Auchenorrhyncha | Tettigometridae | Sap sucker |
| TIN | Hemiptera | Heteroptera | Tingidae | Sap sucker |
| TRO | Hemiptera | Auchenorrhyncha | Tropoduchidae | Sap sucker |
| ANT | Hymenoptera | Apocrita | Anthophoridae | Nectar feeder |
| API | Hymenoptera | Apocrita | Apidae | Nectar feeder |
| BRA | Hymenoptera | Apocrita | Braconidae | Parasitoid |
| CHA | Hymenoptera | Apocrita | Chalcidoidea | Parasitoid |
| CHA 1 | Hymenoptera | Apocrita | Chalcidoidea | Parasitoid |
| CHA 2 | Hymenoptera | Apocrita | Chalcidoidea | Parasitoid |
| CHL | Hymenoptera | Apocrita | Chalcididae | Parasitoid |
| FOR | Hymenoptera | Apocrita | Formicidae | Predator |
| FOR 1 | Hymenoptera | Apocrita | Formicidae | Predator |
| FOR 2 | Hymenoptera | Apocrita | Formicidae | Predator |
| FOR 3 | Hymenoptera | Apocrita | Formicidae | Predator |
| FOR 4 | Hymenoptera | Apocrita | Formicidae | Predator |
| HYM | Hymenoptera | Apocrita | Unknown | Parasitoid |
| HYM 1 | Hymenoptera | Apocrita | Unknown | Parasitoid |
| HYM 2 | Hymenoptera | Apocrita | Unknown | Parasitoid |
| TDA | Hymenoptera | Symphyta | Tenthredinidae | Nectar feeder |
| GEO | Lepidoptera | Glossata | Geometridae | Leaf feeder |
| GEO 1 | Lepidoptera | Glossata | Geometridae | Leaf feeder |
| GEO 2 | Lepidoptera | Glossata | Geometridae | Leaf feeder |
| GEO 3 | Lepidoptera | Glossata | Geometridae | Leaf feeder |
| LEP | Lepidoptera | Glossata | Unknown | Nectar feeder |
| LEP 1 | Lepidoptera | Glossata | Lycaenidae? | Nectar feeder |
| LEP 2 | Lepidoptera | Glossata | Unknown | Nectar feeder |
| LEP 3 | Lepidoptera | Glossata | Unknown | Leaf feeder |
| NOC | Lepidoptera | Glossata | Noctuidae | Leaf feeder |
| NYM | Lepidoptera | Glossata | Nymphalidae | Nectar feeder |
| NYM 1 | Lepidoptera | Glossata | Nymphalidae | Nectar feeder |
| NYM 2 | Lepidoptera | Glossata | Nymphalidae | Nectar feeder |
| NYM 3 | Lepidoptera | Glossata | Nymphalidae | Nectar feeder |
| PIE | Lepidoptera | Glossata | Pieridae | Nectar feeder |
| PSD | Lepidoptera | Glossata | Psychidae | Leaf feeder |
| SPH | Lepidoptera | Glossata | Sphingidae | Leaf feeder |
| MAN | Mantodea |  | Mantidae | Predator |
| MAN 1 | Mantodea |  | Mantidae | Predator |
| MAN 2 | Mantodea |  | Mantidae | Predator |
| MAN 3 | Mantodea |  | Mantidae | Predator |
| MAN 4 | Mantodea |  | Mantidae | Predator |
| CHD | Neuroptera |  | Chrysopidae | Predator |
| CHD 1 | Neuroptera |  | Chrysopidae | Predator |
| CHD 2 | Neuroptera |  | Chrysopidae | Predator |
| MYR | Neuroptera |  | Mymeleontidae | Predator |
| ACR | Orthoptera | Caelifera | Acrididae | Leaf feeder |
| ACR 1 | Orthoptera | Caelifera | Acrididae | Leaf feeder |
| ACR 10 | Orthoptera | Caelifera | Acrididae | Leaf feeder |
| ACR 2 | Orthoptera | Caelifera | Acrididae | Leaf feeder |
| ACR 3 | Orthoptera | Caelifera | Acrididae | Leaf feeder |
| ACR 4 | Orthoptera | Caelifera | Acrididae | Leaf feeder |
| ACR 5 | Orthoptera | Caelifera | Acrididae | Leaf feeder |
| ACR 6 | Orthoptera | Caelifera | Acrididae | Leaf feeder |
| ACR 7 | Orthoptera | Caelifera | Acrididae | Leaf feeder |
| ACR 8 | Orthoptera | Caelifera | Acrididae | Leaf feeder |
| ACR 9 | Orthoptera | Caelifera | Acrididae | Leaf feeder |
| ANO | Orthoptera | Ensifera | Anostostomatidae | Leaf feeder |
| GRY | Orthoptera | Ensifera | Gryllidae | Saprophage |
| GRY 1 | Orthoptera | Ensifera | Gryllidae | Saprophage |
| GRY 2 | Orthoptera | Ensifera | Gryllidae | Saprophage |
| ORT | Orthoptera | Uncertain | Unknown | Leaf feeder |
| PAM | Orthoptera | Caelifera | Pamphagidae | Leaf feeder |
| TET | Orthoptera | Ensifera | Tettigoniidae | Leaf feeder |
| TET 1 | Orthoptera | Ensifera | Tettigoniidae | Leaf feeder |
| TET 2 | Orthoptera | Ensifera | Tettigoniidae | Leaf feeder |
| PHA | Phasmatodea |  | Phasmatidae | Leaf feeder |
| PHA 1 | Phasmatodea |  | Phasmatidae | Leaf feeder |
| PHA 2 | Phasmatodea |  | Phasmatidae | Leaf feeder |
| PSO | Psocoptera |  | Unknown | Saprophage |
| THY | Thysanoptera | Terrebrantia | Thripidae | Sap sucker |
| THY 1 | Thysanoptera | Tubulifera | Phlaeothripidae | Sap sucker |
| THY 2 | Thysanoptera | Terrebrantia | Thripidae | Sap sucker |

Table S3: Information-theoretic model selection process to select the best fit model to explain arthropod abundance.

| Model | Parameters | LogLik | AICc | ΔAICc | Weight |
| --- | --- | --- | --- | --- | --- |
| 1 | Arthropod abundance ~ grass biomass | -57.71 | 129.14 | 0.00 | 0.65 |
| 2 | Arthropod abundance ~ grass height | -59.49 | 132.69 | 3.55 | 0.11 |
| 3 | Arthropod abundance ~grass biomass + insectivore abundance | -56.90 | 133.79 | 4.65 | 0.06 |
| 4 | Arthropod abundance ~ grass biomass + cv grass height | -57.13 | 134.26 | 5.12 | 0.05 |
| 5 | Arthropod abundance ~ grass biomass + tree density | -57.19 | 134.38 | 5.24 | 0.05 |
| 6 | Arthropod abundance ~ intercept only | -62.84 | 134.68 | 5.54 | 0.04 |
| 7 | Arthropod abundance ~ insectivore abundance + grass height | -58.77 | 137.55 | 8.41 | 0.01 |
| 8 | Arthropod abundance ~ tree density + grass height | -58.83 | 137.66 | 8.52 | 0.01 |
| 9 | Arthropod abundance ~ cv grass height + grass height | -59.05 | 138.09 | 8.95 | 0.01 |
| 10 | Arthropod abundance ~ cv grass height | -62.79 | 139.29 | 10.16 | 0.00 |
| 11 | Arthropod abundance ~ tree density | -62.82 | 139.35 | 10.21 | 0.00 |
| 12 | Arthropod abundance ~ insectivore abundance | -62.83 | 139.38 | 10.24 | 0.00 |
| 13 | Arthropod abundance ~ grass biomass + insectivore abundance + cv grass height | -56.22 | 141.24 | 12.10 | 0.00 |
| 14 | Arthropod abundance ~ grass biomass + cv grass height +tree density | -56.69 | 142.18 | 13.04 | 0.00 |
| 15 | Arthropod abundance ~ insectivore abundance + grass height | -58.17 | 145.14 | 16.01 | 0.00 |
| 16 | Arthropod abundance ~ cv grass height | -62.76 | 145.51 | 16.37 | 0.00 |
| 17 | Arthropod abundance ~ insectivore abundance + cv grass height | -62.79 | 145.57 | 16.43 | 0.00 |
| 18 | Arthropod abundance ~ grass height + cv grass height + tree density | -58.41 | 145.61 | 16.47 | 0.00 |

Table S4: Information-theoretic model selection process to select the best fit model to explain arthropod richness.

| Model | Parameters | LogLik | AICc | ΔAICc | Weight |
| --- | --- | --- | --- | --- | --- |
| 1 | Arthropod richness ~ grass biomass | -39.43 | 92.58 | 0.00 | 0.74 |
| 2 | Arthropod richness ~ grass height | -41.45 | 96.61 | 4.02 | 0.10 |
| 3 | Arthropod richness ~grass biomass + cv grass height | -38.75 | 97.49 | 4.91 | 0.06 |
| 4 | Arthropod richness ~ grass biomass + tree density | -39.19 | 98.39 | 5.81 | 0.04 |
| 5 | Arthropod richness ~ grass biomass + insectivore abundance | -39.45 | 98.89 | 6.31 | 0.03 |
| 6 | Arthropod richness ~ grass height + cv grass height | -40.83 | 101.65 | 9.07 | 0.01 |
| 7 | Arthropod richness ~ grass height + insectivore abundance | -41.45 | 102.90 | 10.32 | 0.00 |
| 8 | Arthropod richness ~ grass height + tree density | -41.45 | 102.90 | 10.32 | 0.00 |
| 9 | Arthropod richness ~ grass biomass + cv grass height + tree density | -38.36 | 105.52 | 12.94 | 0.00 |
| 10 | Arthropod richness ~ grass biomass + cv grass height + insectivore abundance | -38.77 | 106.34 | 13.76 | 0.00 |
| 11 | Arthropod richness ~ intercept only | -48.74 | 106.48 | 13.90 | 0.00 |
| 12 | Arthropod richness ~ tree density | -47.73 | 109.17 | 16.59 | 0.00 |
| 13 | Arthropod richness ~ insectivore abundance | -48.33 | 110.38 | 17.79 | 0.00 |
| 14 | Arthropod richness ~ grass height + cv grass height + insectivore abundance | -40.80 | 110.40 | 17.82 | 0.00 |
| 15 | Arthropod richness ~ grass height + cv grass height + tree density | -40.83 | 110.45 | 17.87 | 0.00 |
| 16 | Arthropod richness ~ cv grass height | -48.74 | 111.19 | 18.61 | 0.00 |
| 17 | Arthropod richness ~ cv grass height + tree density | -47.72 | 115.45 | 22.87 | 0.00 |
| 18 | Arthropod richness ~ cv grass height + insectivore abundance | -48.33 | 116.67 | 24.08 | 0.00 |

Table S5: Information-theoretic model selection process to select the best fit model to explain insectivore abundance.

| Model | Parameters | Loglik | AICc | ΔAICc | Weight |
| --- | --- | --- | --- | --- | --- |
| 1 | Insectivore abundance ~ tree density | -43.69 | 96.38 | 0.00 | 0.67 |
| 2 | Insectivore abundance ~ tree density + cv grass height | -43.33 | 100.30 | 3.93 | 0.09 |
| 3 | Insectivore abundance ~ tree density + grass biomass | -43.33 | 100.44 | 4.00 | 0.09 |
| 4 | Insectivore abundance ~ tree density + grass height | -43.64 | 101.00 | 4.62 | 0.08 |
| 5 | Insectivore abundance ~ tree density + arthropod abundance | -43.77 | 101.11 | 4.77 | 0.06 |
| 6 | Insectivore abundance ~ tree density + cv grass height + grass biomass | -42.96 | 105.92 | 9.54 | 0.01 |
| 7 | Insectivore abundance ~ tree density + cv grass height + arthropod abundance | -43.25 | 106.55 | 10.12 | 0.00 |
| 8 | Insectivore abundance ~ tree density + cv grass height + grass height | -43.33 | 106.53 | 10.15 | 0.00 |
| 9 | Insectivore abundance ~ grass height | -58.44 | 125.88 | 29.44 | 0.00 |
| 10 | Insectivore abundance ~ grass biomass | -59.13 | 127.33 | 30.99 | 0.00 |
| 11 | Insectivore abundance ~ cv grass height + grass height | -58.33 | 130.31 | 33.93 | 0.00 |
| 12 | Insectivore abundance ~ cv grass height + grass biomass | -59.02 | 131.88 | 35.44 | 0.00 |
| 13 | Insectivore abundance ~ intercept only | -64.66 | 134.52 | 38.14 | 0.00 |
| 14 | Insectivore abundance ~ arthropod abundance | -64.11 | 137.11 | 40.73 | 0.00 |
| 15 | Insectivore abundance ~ cv grass height | -64.54 | 138.11 | 41.70 | 0.00 |
| 16 | Insectivore abundance ~ cv grass height + arthropod abundance | -64.11 | 141.82 | 45.44 | 0.00 |

Table S6: Information-theoretic model selection process to select the best fit model to explain insectivore richness.

| Model | Parameters | Loglik | AICc | ΔAICc | Weight |
| --- | --- | --- | --- | --- | --- |
| 1 | Insectivore richness ~ tree density | -26.80 | 62.59 | 0.00 | 0.62 |
| 2 | Insectivore richness ~ tree density + arthropod abundance | -25.76 | 65.24 | 2.64 | 0.17 |
| 3 | Insectivore richness ~ tree density + cv grass height | -26.64 | 67.00 | 4.41 | 0.07 |
| 4 | Insectivore richness ~ tree density + grass height | -26.69 | 67.09 | 4.50 | 0.07 |
| 5 | Insectivore richness ~ tree density + grass biomass | -26.77 | 67.25 | 4.66 | 0.06 |
| 6 | Insectivore richness ~ tree density + cv grass height + arthropod abundance | -24.98 | 69.96 | 7.37 | 0.02 |
| 7 | Insectivore richness ~ tree density + cv grass height + grass height | -26.50 | 72.99 | 10.40 | 0.00 |
| 8 | Insectivore richness ~ tree density + cv grass height + grass biomass | -26.60 | 73.20 | 10.61 | 0.00 |
| 9 | Insectivore richness ~ grass height | -35.95 | 80.90 | 18.31 | 0.00 |
| 10 | Insectivore richness ~ intercept only | -38.48 | 82.30 | 19.70 | 0.00 |
| 11 | Insectivore richness ~ grass biomass | -36.86 | 82.72 | 20.13 | 0.00 |
| 12 | Insectivore richness ~ cv grass height + grass height | -35.94 | 85.59 | 23.00 | 0.00 |
| 13 | Insectivore richness ~ arthropod abundance | -38.32 | 85.64 | 23.05 | 0.00 |
| 14 | Insectivore richness ~ cv grass height | -38.47 | 85.93 | 23.34 | 0.00 |
| 15 | Insectivore richness ~ cv grass height + grass biomass | -36.85 | 87.41 | 24.81 | 0.00 |
| 16 | Insectivore richness ~ cv grass height + arthropod abundance | -38.22 | 90.16 | 27.57 | 0.00 |

**REFERENCES**

Bibby, C. J. (Ed.). (2000). *Bird census techniques*, 2nd ed, London ; San Diego: Academic.

Colwell, R. K., & Coddington, J. A. (1994). Estimating terrestrial biodiversity through extrapolation. *Philosophical Transactions of the Royal Society of London. Series B: Biological Sciences*, **345**(1311), 101–118.

Hockey, P., Dean, W. Richard J., & Ryan, P. (2006). *Roberts birds of southern Africa*.

Kemp, A. C., Herholdt, J. J., Whyte, I. J., & Harrison, J. (2001). Birds of the two largest national parks in South Africa : a method to generate estimates of population size for all species and assess their conservation ecology : research article. *South African Journal of Science*, **97**, 393–403.

Picker, M., Griffiths, C, & Weaving A. (2019). Field Guide to Insects of South Africa. Third Edition. Struik Nature (Random House Struik), Cape Town, South Africa.

R Core Team. (2021). R: A language and environment for statistical computing, R Foundation for Statistical Computing. Retrieved from https://www.R-project.org/

Ralph, C. J., Sauer, J. R., & Droege, S. (1995). *Monitoring bird populations by point counts* (No. PSW-GTR-149), Albany, CA: U.S. Department of Agriculture, Forest Service, Pacific Southwest Research Station. Retrieved from https://www.fs.usda.gov/treesearch/pubs/31461

Robinson, T. B., Branch, G. M., Griffiths, C. l., Govender, A., & Hockey, P. A. R. (2007). Changes in South African rocky intertidal invertebrate community structure associated with the invasion of the mussel Mytilus galloprovincialis. *Marine Ecology Progress Series*, **340**, 163–171.
